# Evaluation of the effects of a designated program on illegal drug cessation among adolescents who experiment with drugs

**DOI:** 10.1186/s13011-017-0139-9

**Published:** 2018-01-16

**Authors:** Chiu-Ching Chang, Jung-Yu Liao, Chiu-Mieh Huang, Hsiao-Pei Hsu, Chih-Che Chen, Jong-Long Guo

**Affiliations:** 1 Department of Health Promotion and Health Education, National Taiwan Normal University, No. 162, Section 1, Heping East Road, 10610 Taipei, Taiwan; 2 Institute of Clinical Nursing, National Yang-Ming University, No. 155, Section 2, Li-Nong Street, Taipei, Taiwan 11221; 3 Department of Health Promotion and Health Education, College of Education, National Taiwan Normal University, No. 162, Section 1, Heping East Road, 10610 Taipei, Taiwan

**Keywords:** Adolescent, Illegal drug use, Learning climate, Intervention study, Vocational high school

## Abstract

**Background:**

Studies indicate that adolescent-onset drug users experience a greater likelihood of dependence that continues into adulthood. The importance of early intervention was evident in treating adolescents before their substance use progressed. We examined the effectiveness of an intervention program that prevents students who experiment with drugs from reusing them.

**Methods:**

The study was based on 10 out of 18 invited schools that were randomly assigned to either the intervention group (5 schools, *n* = 43) or the comparison group (5 schools, *n* = 41). The intervention group received an E-course program that comprised a main intervention course (12 sessions) and a booster course (2 sessions). By reducing the burden of teaching content during the 14 sessions, the in-class counselor had opportunities for face-to-face discussions with students on their ambivalence toward quitting illegal drugs. The comparison group received the conventional didactic drug prevention course (2 sessions). Outcomes in terms of stress management, refusal skills, pros of drug use, cons of drug use, and drug use resistance self-efficacy were measured via structured questionnaires conducted thrice: at baseline, after the main intervention sessions, and after the booster sessions. A linear mixed model (LMM) was employed to investigate the effects of time and groups on the outcome variables with group, time, and group × time as fixed effects. Subjects and schools were selected as random effects in order to consider both within-subject and within-school correlations.

**Results:**

There was a significant group × time interaction with regard to stress management, refusal skills, pros of drug use, and drug use resistance self-efficacy, excluding cons of drug use. The intervention group displayed better stress management compared to the comparison group after the booster intervention. Similar between-group differences were identified in that the intervention group displayed better refusal skills and drug use resistance self-efficacy compared to that of the comparison group. The intervention group favored using drugs less (a decrease in the pros of drug use score) compared to the comparison group after the booster intervention.

**Conclusions:**

Our program provided an example of the results of early intervention among students who experiment with illegal drugs.

## Background

Drug use prevalence identified in Taiwan is relatively lower than that reported in Monitoring the Future, the U.S. survey [1], in which lifetime prevalence of using any illegal drugs among adolescents (12th grade) ranged from 40% to 55%. The prevalence among vocational students is higher than among full-time students in Taiwan. The lifetime prevalence of illegal drug use among high school and vocational high school students was 0.27% [2] and 2.3–2.7%, respectively [3, 4]. The prevalence of experimental and regular illegal drug use among vocational high school night-class students was 13.6% and 4.7%, respectively [5].

Adolescents who experiment with drugs put their health and safety at risk. The short-term effects of drug use can affect a person’s thinking, mood, energy level, and perception. They may impair motor functioning, interfere with decision making and problem solving, as well as cause physical health problems [6, 7]. Chronic and persistent drug use may lead to the development of an addiction and can be associated with interferences with work and school and suspension or expulsion from school [6, 7]. Adolescence is a critical transition period of vulnerability for the onset of illegal drug use, as early use of drugs increases the chances of developing addictions [8, 9]. Studies have shown that adolescent-onset drug users experience a greater likelihood of dependence and more serious clinical syndromes than adult-onset drug users [10].

Unlike full-time high school students, most vocational high school students are required to spend time at a workplace as part of their education program. Occasionally, the training environment outside school communicates an acceptable norm for smoking and drinking, making it more likely for the students to be exposed to a pro-substance use environment [11]. The use of tobacco or alcohol has been shown to be associated with a greater likelihood of illegal substance use [5]. The use of illegal drugs leads to a number of adverse effects, including physical and mental disorders, rule-breaking behavior, unintended injuries, violence, poor academic performance, and dropping out of school [5, 8, 12]. Therefore, it is important to develop an effective program for assisting vocational high school students who experiment with illegal drugs to avoid drug use.

Evidence-based interventions are effective in helping prevent drug use among at-risk adolescents [13]. When young people perceive drug use as harmful, they reduce their level of use [14]. Social influence of drug use [15], decisional balance [16], and drug use resistance self-efficacy [17] have been found to be effective components of drug prevention programs. Decisional balance is important in changing motivations and involves assessing the pros and cons of changes in behavior before embarking on behavioral changes [18]. The greater an individual’s efficacy, the more likely it is for that person to adopt the desired healthy behavior [19]. In addition to being cognitively prepared for not using drugs [20], students need to be taught the skills needed to resist the social pressure to use these drugs and to manage the stresses associated with the risk of continued drug use. The World Health Organization (WHO) defined life skills as “abilities for adaptive and positive behavior that enable individuals to deal effectively with the demands and challenges of everyday life” [21]. Life skill helps build up a skills’ repertoire that allows students to deal with situations of drug use temptation. They also help students translate attitudes into actions, thereby increasing their self-efficacy of drug use resistance and ability to deal with social influence [22]. Consequently, substance use behavior decreases while life skills are fostered [23].

As mentioned above, many research studies have examined the effectiveness of empirically supported learning contents for drug use prevention. Relatively, less attention has been paid to learning climate, an emotional atmosphere that determines learning, and the progress made by each student [24]. Learning climate has been shown to affect learners’ motivation and self-confidence, and it can increase success levels and decrease anti-social behavior [24]. A previous study found that as teachers’ interest in a positive school climate increases, the percentage of students who use drugs decreases [25]. Teachers can help build respectful school climates that support and promote interaction and mutual respect. Teachers’ empathy and appreciation of a student’s uniqueness were also related to positive changes in motivation and engagement, which had an impact on learning climate [26].

Failure to abstain from drug use may be attributed to lack of motivation. A positive classroom climate has been shown to be related to motivation for adopting expected behavior [27]. Therefore, we created a classroom in which the counselor and students worked together to create and sustain an environment in which everyone felt safe, supported, and encouraged to express their views and concerns. The purpose of this study was to examine the effectiveness of a counselor-guided drug abstinence program for vocational high school students who experiment with drugs.

## Methods

### Participants and procedures

With the help of school personnel, we invited the vocational high schools that had students who currently experimented with illegal drugs, which was verified by a urine test. This study was based on 10 out of 18 invited schools whose principals and responsible personnel agreed to participate in the study. The 10 schools were randomly assigned to the intervention and comparison groups by drawing lots (five schools in each group); therefore, students of these two groups came from different schools. Because of the scarce number of illegal drug-use students who met the study inclusion criteria, non-probability school sampling was used.

The research team visited the schools and presented the program to the principals and other responsible personnel. Program materials were demonstrated and discussed during the meetings. The selection criteria included (a) currently enrolled students with drug use experience, which was verified by a urine test, (b) students’ willingness to comply with verbal and written instructions in the computer classroom, (c) students’ ability to use mobile phones, (d) no existing cognitive impairment or learning disabilities among students and (e) students’ willingness to participate in the study and provide a signed informed consent form. After obtaining consent, participants in the intervention group underwent the counselor-guided program on illegal drug cessation, while those in the comparison group received conventional didactic education related to illegal drug use. The preventive program on illegal drug reuse included a main intervention course (12 sessions held over approximately three months) and a booster course (2 sessions held over a month). In this study, the intervention and comparison groups were compared at the baseline (T1), after the main intervention course (T2), and after the booster course (T3).

Using power tables as suggested by Stevens [28], a sample size of 39 students per group was required to achieve a power of 0.8 and a medium effect size of 0.3 for measurements repeated three times at an α level of 0.05.

### Program design

#### Learning climate

This program emphasized the construction of a safe and supportive learning climate to enhance students’ sense of trust and belonging by relationship building, exchange of expectations, empathy and support, and appreciation of an individual’s uniqueness. The feature that distinguished our program from previous studies was that we emphasized on students’ learning instead of teachers’ instruction. To reduce the burden of instruction, learning contents were delivered in a computer classroom. Each participant received an anonymous ID and password to log in and learn at each session.

The counselor played the vital role of facilitating learning, solving problem, leading discussions, and providing feedback. A primary counselor collaborated with responsible personnel from schools to provide assistance to the students. She is a registered nurse, a PhD candidate specialized in health education and health behavior, and an instructor of the Train the Trainer Conference for campus drug prevention. The counselor made great efforts for developing a safe and supportive learning climate. Before or/and after course sessions, the course counselor initiated various activities such as “nice to meet you,” “what we can do while we are together,” “you are not alone,” and “you can make it.” “Nice to meet you” was an introduction of the counselor to participating students in order to build cooperative relationships. The counselor encouraged participating students to type down their expectations under their accounts for “What we can do while we are together.” In response to those students who revealed their barriers to stopping drug use, the counselor accompanied them and provided them with empathy and support in “you are not alone.” “You can make it” involved appreciations from the counselor to affirm students’ motivation to change.

The counselor guided the participants to learn through the designed learning activities. We created a classroom in which the counselor and students worked together to establish and sustain an environment in which everyone felt safe, supported, and encouraged to express their concerns.

The concept of coupon incentives comes from the financial incentives shown to enhance participation in abstinence programs. Coupons not only function as financial incentives but also as a positive climate to recognize students’ achievement [29]. To honor their efforts, participants who completed the illegal drug cessation program received a formal certificate at the closing ceremony. To emphasize the positive changes, we used coupons from convenience stores as rewards for their efforts to resist drug use. A urine test was administrated only to participants who were willing to accept the test as a confirmation of drug abstinence. Those who passed the urine test received a coupon worth NT$200 (approximately $7 US dollars).

#### Learning contents

**Fig. 1:**
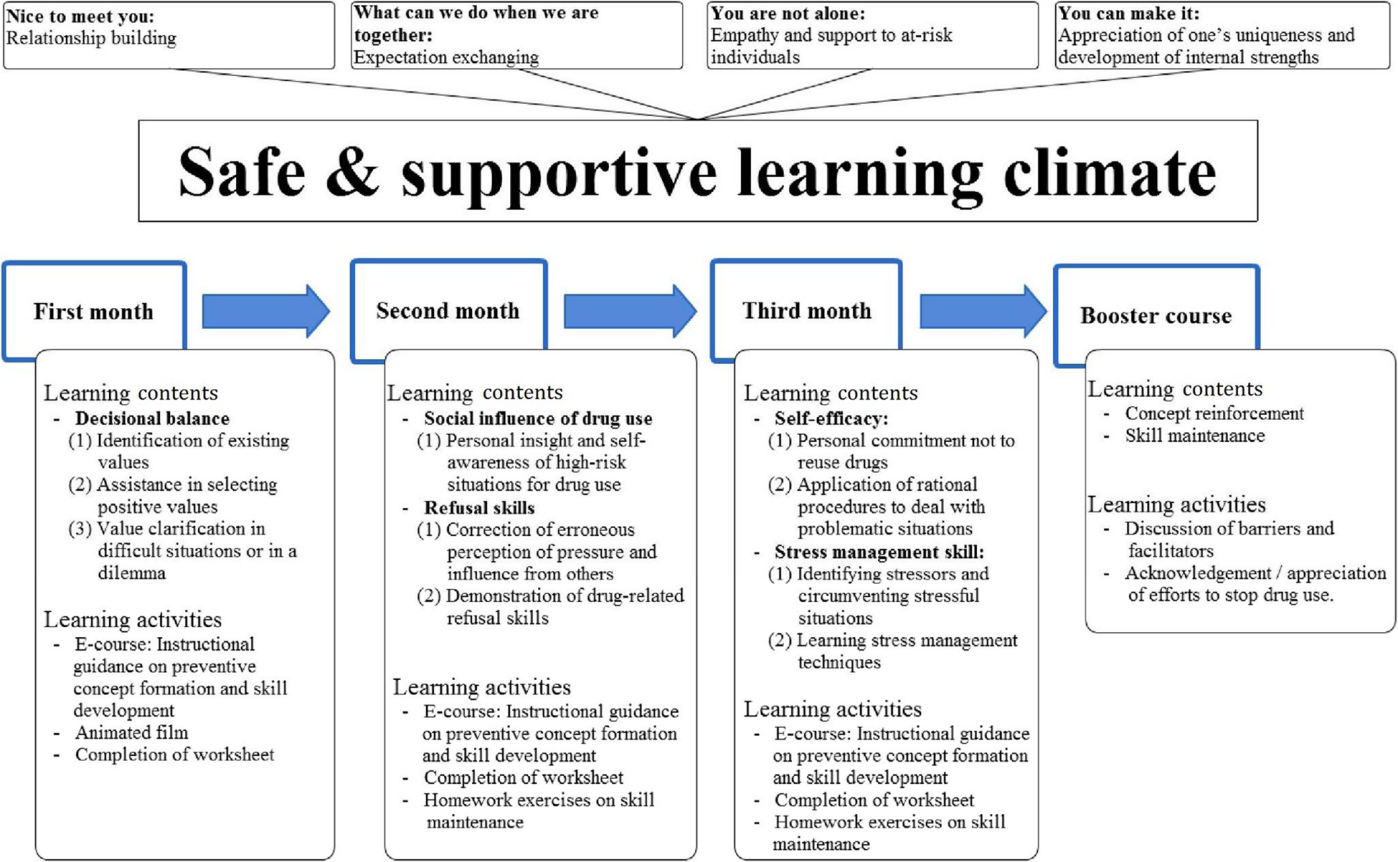
Intervention program developed by the research team

The learning contents of the proposed program were derived from previous empirical research and theory literature (Fig. 1). The learning contents comprised two parts, preventive concepts formation and skill development. The components related to decisional balance, social influence of drug use, and self-efficacy of nonuse were adopted for preventive concepts formation. The contents of decisional balance included identifying the causes of drug use, health hazards of drug use, and myth clarification to assist students’ decision wisely. The contents of social influence included awareness of drug use–induced situations to enhance students’ insight related to high-risk situations. The contents of self-efficacy included a demonstration of positive self-talking, commitment to not reuse drugs, and a storytelling film on positive role models to increase students’ confidence. Skill development included two components of stress management and refusal skills. The contents of stress management included identifying stressors, a demonstration of stress-relieving techniques, and a demonstration of alternative health activities. The contents of refusal skills included examples of demonstrating refusal strategies and example sentences of refusing drugs while faced with temptation. Students were encouraged to review the contents of the main course during the booster intervention. No extra contents other than those in the main course were added. Students were welcome to express their personal positive or negative experiences related to avoiding drug use.

The main intervention course comprised twelve 90-min sessions delivered in the fall semester, and the booster course comprised two 90-min sessions delivered in the spring semester. The booster course was performed approximately three months after completion of the main intervention course to reinforce preventive concepts, maintain skills, and help sustain the intervention effectiveness.

During the study, we applied text messaging using mobile phones to extend the connection with participants in the intervention group outside the classroom. Each participant was sent 12 messages during the period of study. Text messages were sent out after the first session with about one week’s interval per message. These messages were developed by a research team as reinforcement for encouraging zero drug-use and preventing reuse. There were two kinds of text messages included, namely, cue to action and social support. For example, a cue to action text message stated, “Look at the cons of using illegal drug, display your specific reasons of staying drug-free where you can see them.” “Believe in yourself, we are all here to support you to quit illegal drug use” is an example text message for social support.

### Outcome measures

Assessments were made using a structured self-report questionnaire in Chinese that was developed and modified based on a literature review. The questionnaire included items on demographic information, illegal drug use, skill-learning, and cognitive status.

The measures for participants’ demographics included age (years), gender (male/female), work status of main guardian (employed/ unemployed), years of education of main guardian (< 9, ≥ 9 years), household status (live with both parents, live with single parent, live without parent), religious belief (yes, no), and illegal drug use among peers (yes, no), as listed in Table 1.

**Table 1:** Comparisons of the intervention and comparison groups at baseline

Characteristics	Intervention (*n* = 43)	Comparison (*n* = 41)	t/χ^2^	*p* value
Age (years)	17.14 ± 0.91	17.43 ± 0.14	*t* = 2.11	0.15
Gender			χ^2^ = 0.34	0.56
Male	29 (67.44)	31 (75.61)		
Female	14 (32.56)	10 (24.39)		
Work status of main guardian			χ^2^ = 0.03	0.86
Employed	34 (79.07)	34 (82.93)		
Unemployed	9 (20.93)	7 (17.07)		
Years of education of main guardian			χ^2^ = 0.86	0.36
≤ 9 years	12 (27.91)	7 (17.07)		
>9 years	31 (72.09)	34 (82.93)		
Household status			χ^2^ = 0.92	0.34
Live with both parents	27 (62.79)	30 (75.00)		
Others	16 (37.21)	10 (25.00)		
Religious belief ^a^			χ^2^ = 0.31	0.58
Yes	18 (43.90)	21 (52.50)		
No	23 (56.10)	19 (47.50)		
Illicit drug use among peers			χ^2^ = 1.14	0.29
Yes	29 (67.44)	22 (53.66)		
No	14 (32.56)	19 (46.34)		
Outcome measures at baseline^b^			T^2^ = 8.00 (F = 1.50)	0.20
Stress management	32.29 ± 5.49	33.02 ± 5.47		
Refusal skills	26.00 ± 4.32	26.52 ± 4.11		
Pros of drug use	21.63 ± 10.37	18.82 ± 12.81		
Cons of drug use	37.67 ± 9.00	36.53 ± 12.17		
Drug use resistance self-efficacy	31.55 ± 5.51	34.14 ± 4.01		

Data of participants’ characteristics are presented as mean, standard deviation, or number (percent) and were compared by an independent two-sample t-test or the chi-square test with Yates correction

^a^Not all students provided complete answers

^b^Differences of outcome measures between groups at baseline were compared by Hotelling’s T^2^

The measures of illegal drug use were urine drug testing. The measures of skill learning were stress management and refusal skills. The measures of cognitive status included decisional balance (pros and cons of use) and drug use resistance self-efficacy.

#### Measures of illegal drug use: Urine test

A urine test is a common method to verify abstinence from illegal drug use. We used it to confirm self-reported abstinence. The test results were judged by the Enzyme-Multiplied Immunoassay Technique (EMIT) or by Fluorescence Polarization Immunoassay (FPIA) [30]. The cutoffs for a positive result are 500 ng/ml for amphetamines, 300 ng/ml for MDMA (ecstasy), 100 ng/ml for ketamines, and 50 ng/ml for marijuana.

#### Measures of skills learning: Stress management

The Perceived Stress Scale (PSS) is a widely used psychological instrument for measuring experienced stress [31, 32]. Items were designed to capture respondents’ appraisal about how unpredictable, uncontrollable, and overloaded their lives were. We adopted the scores of PSS to represent the participants’ ability to manage stressful situations. Responses on a 5-point Likert-type scale ranged from 1 (never) to 5 (very often). The scale included four positively stated and six negatively stated items. PSS scores were obtained by reversing responses to the six negatively stated items. The scores were obtained by summing across all scale items. A higher score indicated better stress management. The Cronbach’s α was 0.79.

#### Measures of skills learning: Refusal skills

The scale of drug refusal skills was assessed using six items that measure the likelihood of practicing various refusal techniques while participants were offered drugs, on a 5-point Likert-type scale ranging from 1 (completely impossible) to 5 (completely possible). There was no reverse scoring item in this scale. The scale items were derived from the examples illustrated in the E-course. Some sample items included “finding excuses to leave,” “promise someone (e.g., parents or boy/girlfriend) not to use drug,” and “just say no.” The scores were obtained by summing across all scale items. A higher score indicated greater probability of practicing refusal skills. The Cronbach’s α was 0.83.

#### Measures of cognitive status: Pros and cons of drug use

The decisional balance was measured using a method adopted from a previous study [33]. There were 10 statements each for measuring the pros and cons of drug use. The participants rated the advantages and disadvantages of using drugs. They were asked to rate the level of importance as the statements related to the decision making about drug use. Each item on this 5-point Likert-type scale was scored from 1 (not important) to 5 (extremely important). For the pros, the scores were obtained by summing across all scale items, and a higher score indicated that the students tended to favor using drugs. The sample item was “Drug use helps me to loosen up and express myself.” Cronbach’s α was 0.97. Similarly, for the cons, the scores were obtained by summing across all scale items, and a higher score on the cons indicated that the participants tended to avoid using drug. The sample item was “My drug use causes problems with others.” The Cronbach’s α was 0.93.

#### Self-efficacy of illegal drug use resistance

Self-efficacy is defined as situation-specific confidence that people can cope with high-risk situations without relapsing into unhealthy behavior [34]. In this study, nine descriptions were developed to address the situations wherein students may encounter temptations. Situation-specific descriptions were derived from the Drug Use Resistance Self-Efficacy (DURSE) scale [35]. The DURSE measured the level of self-efficacy of drug use resistance if friends, family, siblings, or relatives offered illegal drugs under specific situations or places such as parties, homes, and social gathering places. The 4-point Likert-type scale ranged from 1 (not sure at all) to 4 (definitely sure). The sample item was “How sure are you that you can refuse if a friend offers you illegal drugs at a party.” The scores were obtained by summing across all scale items. A higher score indicated a greater drug use resistance self-efficacy. The Cronbach’s α was 0.80.

### Statistical analysis

Statistical analyses were performed using SPSS 22.0 (SPSS Inc., Chicago, IL). Categorical variables were presented as counts and percentages. Continuous variables were presented as mean and standard deviation. Differences between the intervention and the comparison groups were compared by chi-square test with Yates correction (categorical variables). Yates’ correction was suggested because not all expected counts were 10 or greater for the 2*2 Table [28]. Because the use of a fragmented univariate test may lead to inflated overall type I error, Hotelling’s T2 (multivariate two-group test) was performed for group comparisons of the five outcome measures at baseline [28]. A linear mixed model (LMM) was used to investigate the effects of time and groups on the outcome variables. The LMM was performed with group, time, and group × time as fixed effects. Subjects and schools were selected as random effects in order to take into consideration both within-subject and within-school correlations. Missing data are common in repeated-measure designs. LMMs can accommodate all of the data that are available for a given subject without disregarding any of the data collected. The patterns of change and the effects at both the individual and group levels were to be understood by LMM [36].

### Ethical considerations

The study protocol was approved by the research ethics committee of National Taiwan University. After students’ experiences of drug use were verified by a urine test, school personnel were required to contact their guardians according to the official guidelines. Families play an important role in cooperating with school personnel to assist students’ drug cessation. However, in some cases, families are contributors to students’ drug use. We, along with the school personnel, made several attempts to facilitate parents/guardians to get involved in the students’ cessation process but were frustrated by their lack of response. To protect underage students, alternative strategies were provided to deal with the difficulty, including fully respecting students’ willingness to participate, provision of an information letter, making available a telephone number for parents/guardians to ask questions or express their opinions (e.g., reject their children’s participation), and ensuring that at least one school personnel was present at each E-course session. The information letter including the study purpose, course description, and a telephone number for “Q&A” were given to students to take home for their main guardian. Guardians and students were welcome to raise any concerns and questions related to the study. Only the participants who signed an informed consent form took part in the study. The participants were permitted to withdraw from the study at any point, if they wished to do so, without affecting their academic score. The outcome measures were identified only by numbers; therefore, participant identity and the gathered information were not associated, thereby ensuring anonymity.

## Results

### Participant characteristics

**Fig. 2:**
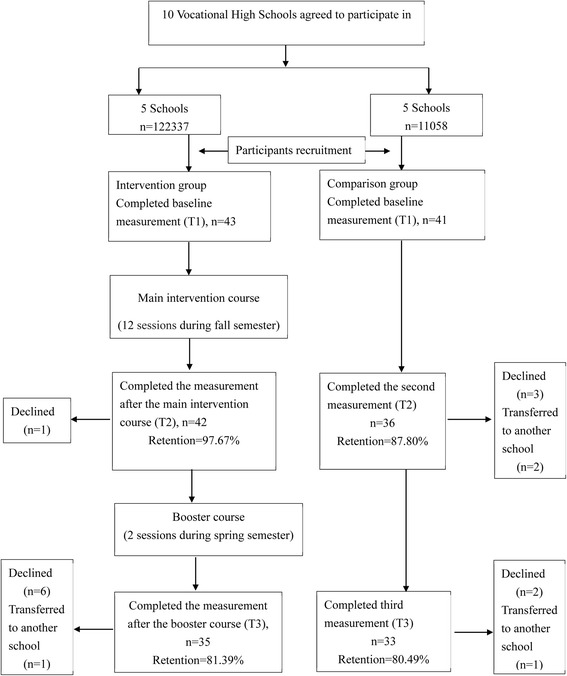
Study flow diagram

We recruited 43 and 41 students for the intervention and comparison groups, respectively; a total of 84 students enrolled in the study. After the booster course, 35 and 33 students remained in the intervention and comparison groups, respectively. The reasons for declining participation included being no longer interested in participation, unavailability of time, transfer to another school, or absence without reasons. The attrition rates were lower than 20% both in the intervention and comparison groups. Figure 2 depicts a flowchart displaying participants’ enrollment and assessments.

The distribution and comparisons of participant characteristics in the two groups are summarized in Table 1. The intervention and comparison groups were similar in age (17.14 ± .91 years and 17.43 ± .14 years, respectively) and gender. No statistically significant differences were observed between the two groups in terms of age, gender, work status of main guardian, years of education of main guardian, household status, and religious belief. It indicated that the participants of both groups were similar in demographic characteristics. The group comparisons of outcome measures at baseline were determined by performing Hotelling’s T^2^. The results revealed that the five outcome measures at baseline were not statistically different between groups (T^2^ = 8.00, F = 1.5, d.f. = 5, 78; *p* = 0.2). The analyses were performed overall to avoid inflating type I error. Since the overall result revealed non-statistical significance, we did not analyze each scale separately.

### Outcome measures

#### Urine test

The urine test results of all participants were positive before attending this study (one of the selection criteria). The urine test results among all participants of the intervention group indicated negative responses after the main intervention (T2) as well as the booster intervention (T3), which demonstrated that these participants consistently avoided using illegal drugs. However, two participants of the comparison group showed positive results after the booster intervention (T3). No inferential statistics were performed to compare the group difference since many cell counts were zero.

#### Skill and cognitive variables

**Fig. 3:**
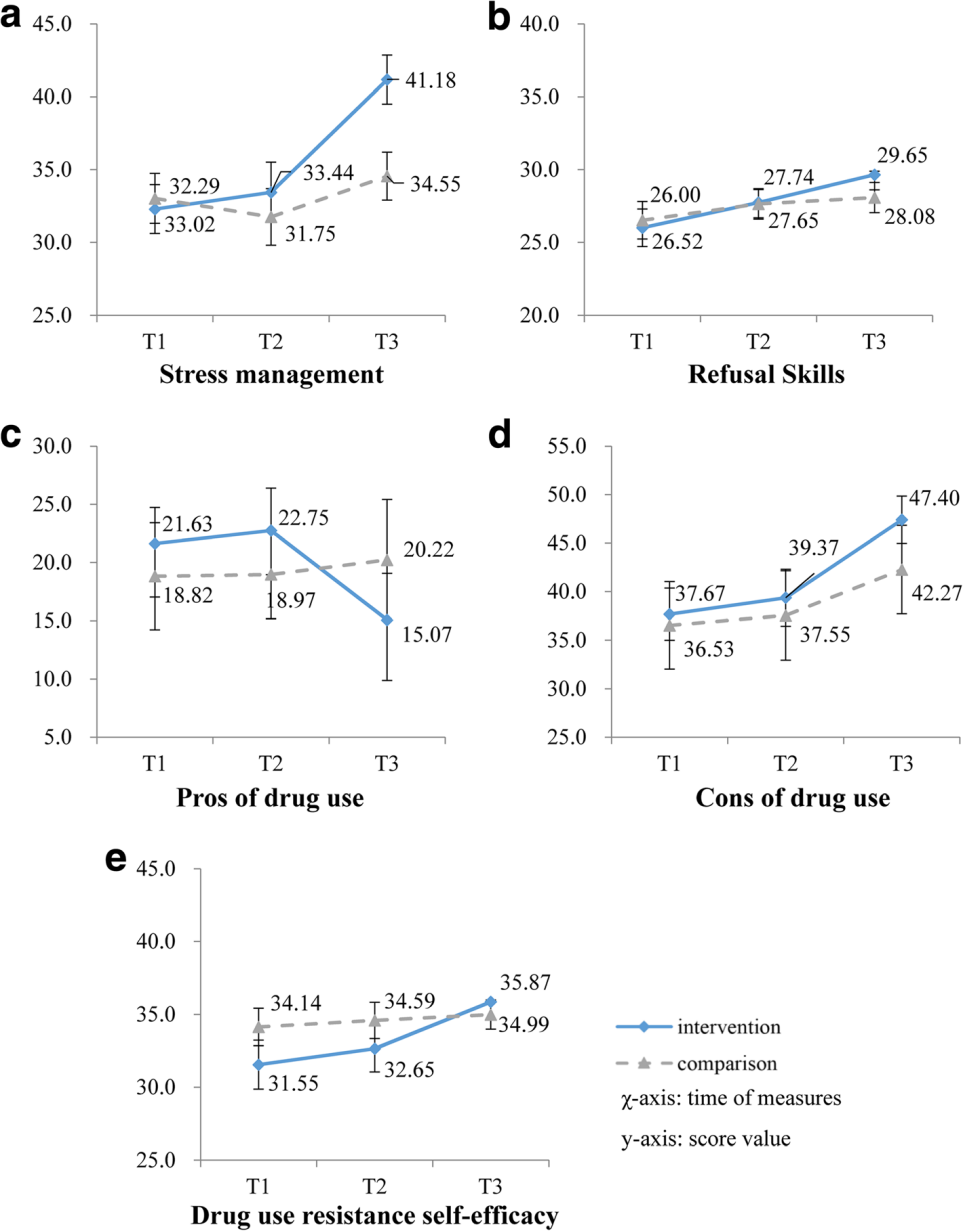
Outcome measures of stress management (**a**), refusal skills (**b**), pros of drug use (**c**), cons of drug use (**d**) and drug use resistance self-efficacy (**e**) in the intervention and comparison groups from baseline (T1) to the end of the main intervention (T2) and after the booster intervention (T3)

Figure 3 summarizes the changes in two skills (stress management and refusal skills) and three cognitive statuses (pros and cons of drug use and drug use resistance self-efficacy) for the two groups. LMMs were used to examine the group differences in patterns of change over time (Table 2). Results of the LMM analysis showed that the intervention group made nonsignificant improvements compared to the comparison group after the main intervention in terms of the scores of stress management (β =2.41, *t* = 1.81, *p* = 0.073), refusal skills (β =0.61, *t* = 0.62, *p* = 0.534), pros of drug use (β =0.97, *t* = 0.38, *p* = 0.703), cons of drug use (β =0.68, *t* = 0.25, *p* = 0.802) and drug use resistance self-efficacy (β =0.64, *t* = 0.51, *p* = 0.609). However, after the booster intervention, the participants of the intervention group showed significant improvements compared to their counterparts in the comparison group. There was a significant group × time interaction for the four outcome measures except for cons of drug use (β =3.98, *t* = 1.45, *p* = 0.150). The intervention group showed an increase in the score of stress management as compared to the comparison group after the booster intervention (β =7.35, *t* = 5.19, *p* < 0.001). Similar patterns of between-group differences were found in the scores of refusal skills and drug use resistance self-efficacy (β =2.09, *t* = 2.10, *p* = 0.038; β =3.47, *t* = 3.15, *p* = 0.002). The intervention group showed a decrease in the score of pros of drug use as compared to the comparison group after the booster intervention (β = −7.96, *t* = −2.62, *p* = 0.010).

**Table 2:** Liner mixed model analysis of outcome measures

	Regression coefficient	Standard error	*t*-value	*p* value
Stress management				
Group (intervention) ^a^	−0.79	1.55	−0.51	0.619
Time (T2) ^b^	−1.27	1.22	−1.04	0.299
Time (T3) ^b^	1.54	1.08	1.42	0.157
Group (intervention) × Time (T2) ^c^	2.41	1.70	1.42	0.159
Group (intervention) × Time (T3) ^c^	7.35	1.51	4.87	<0.001
Refusal skills				
Group (intervention) ^a^	−0.63	1.00	−0.63	0.532
Time (T2) ^b^	1.13	0.78	1.44	0.151
Time (T3) ^b^	1.56	0.71	2.20	0.030
Group (intervention) × Time (T2) ^c^	0.61	1.09	0.56	0.577
Group (intervention) × Time (T3) ^c^	2.09	0.99	2.10	0.038
Pros of drug use				
Group (intervention) ^a^	2.51	3.14	0.80	0.433
Time (T2) ^b^	0.15	2.42	0.06	0.950
Time (T3) ^b^	1.41	2.36	0.59	0.553
Group (intervention) × Time (T2) ^c^	0.97	3.38	0.29	0.774
Group (intervention) × Time (T3) ^c^	−7.96	3.31	−2.41	0.017
Cons of drug use				
Group (intervention) ^a^	1.35	2.44	0.55	0.586
Time (T2) ^b^	1.01	2.39	0.43	0.671
Time (T3) ^b^	5.74	2.09	2.75	0.007
Group (intervention) × Time (T2) ^c^	0.68	3.34	0.20	0.838
Group (intervention) × Time (T3) ^c^	3.98	2.92	1.37	0.174
Drug use resistance self-efficacy				
Group (intervention) ^a^	−2.58	1.06	−2.45	0.017
Time (T2) ^b^	0.45	1.02	0.45	0.656
Time (T3) ^b^	0.85	0.80	1.06	0.292
Group (intervention) × Time (T2) ^c^	0.64	1.42	0.45	0.652
Group (intervention) × Time (T3) ^c^	3.47	1.12	3.09	0.003

T1: Baseline, T2: The end of the main intervention, T3: After the booster intervention

^a^Reference group: Comparison group

^b^Reference group: T1

^c^Reference group: Group (comparison) × Time (T1)

Liner mixed model was performed with group, time and group × time as fixed effects and subjects and schools as random effects

## Discussion

We used a repeated-measure design to evaluate the effectiveness of the intervention program. It is noteworthy that the effects on improving skills and cognitive measures were significant after the booster intervention, but not evident after the main intervention. Guo, Lee, Liao and Huang [37] suggested that booster courses increased the effectiveness of an intervention by providing the opportunity to build on and reinforce desired messages and skills. Congruent with previous studies, the findings of this study provided empirical evidence to support the necessity of arranging a booster course for students who experiment with drugs.

In terms of the length of the intervention, the findings of a previous meta-analysis suggested that early interventions do not need to be lengthy to be effective with adolescents [38]. However, the review of brief interventions (defined as up to four sessions in length) on substance-using adolescents (with a mean age of 16.9 years) suggested that brief interventions did not have a significant effect on substance use outcomes [39]. The length of our program was 14 sessions including the main intervention (12 sessions) and the booster intervention (2 sessions). Judgment of an appropriate intensity of intervention needs further investigation.

Our program worked with adolescents who were at the stage of experimenting with drugs, but who did not meet the criteria for substance use disorder. A previous study defined the intervention targeting adolescents who experiment or initiate drugs as *early intervention* [40]. Experimental use may not lead invariably to deleterious patterns of use. However, the results of a meta-analysis study revealed strong evidence in support of the effectiveness of early interventions for adolescent substance use [39]. The importance of early intervention was evident for treating adolescents before their substance use progressed and before they needed any special treatment [39]. Our program provided an example of early intervention for the students who were screened out as current illegal drug users.

Our program contents were similar to the Life Skills Training (LST) program, which is empirically supported as an effective prevention program for adolescent drug abuse and is also a kind of early intervention [40]. Many adolescent drug users are reluctant to quit when faced with situational temptation [21]. LST emphasizes on teaching youth how to recognize and resist pressures to use drugs and promote anti-drug use norms. It also combines social resistance skills training with competence enhancement skills building [40]. The skill learning and cognitive improvements help dissuade them from continuing the use of illegal drugs and help motivate them to quit this habit [16, 22, 23, 37].

Due to time constraints and man power limitations, our program could not afford an intervening intensity as high as the LST program suggested. The LST program is implemented across 15 classes (about 45 min each) in grade seven, 10 booster sessions in grade eight, and 5 booster sessions in grade nine. The LST program is taught using cognitive-behavioral skills training techniques and facilitates group discussions, classroom demonstrations, and traditional didactic teaching methods. The novelty of our program is that learning contents were delivered on an E-course-basis, thus helping reduce the teaching burden of the counselor. The participants were also provided with the chance to learn at their own pace. Adolescents who are early drug users need a learning climate that promotes prosocial engagement. The in-class counselor had opportunities for face-to-face discussions on their ambivalence toward quitting illegal drugs; this is a necessary process to foster confidence in reluctant participants. The counselor encouraged participants to express their expectations of this program. A discussion about barriers and facilitators of stopping drug use prompted the participants to make positive decisions. Participants were encouraged to develop individualized cessation strategies and were provided with counselor support to help them execute the said strategies. In order to extend a supportive climate outside the classroom, mobile phone text messages were sent to participants with behavioral cues and support. Text messages have proven to be an effective strategy to help quit drug abuse [16]. Thus, we constructed a climate for fostering participants’ successful experiences. Coupon-based incentives have also been shown to reinforce the achievements of students in drug quitting behavior [41]. The urine test validated participants’ achievement of drug cessation. However, it was not welcome by participants at the beginning because they did not want to be a “suspect” and under “monitoring.” We hosted a discussion to explore the positive “meaning” of taking a urine test. A new concept related to urine tests emerged. The urine test became a reference for the success of the participants, who visualized it as their achievement, instead of being a “monitoring” measure for suspecting adolescents with a drug use problem. Some students’ provided feedback that it was the first time they received a certificate and reward for their achievement.

We attribute the effectiveness of our program to its comprising evidence-based preventive strategies and to its administration in a safe and supportive learning climate. A positive learning climate is an important part of staging effective interventions, although the program’s effects are not limited to climate-driven changes alone. We did not directly measure the responses of the students in the intervention group on learning climate since both groups received the same quantitative measurements. The students of the intervention group were encouraged to describe their thoughts and feelings about each session (through an open question). Most of the retention students of the intervention group automatically provided written responses for more than six sessions (29/35 = 83%).The mean of word counts from 23.82 (SD = 17.86) during the first session to 39.07 (SD = 36.74), excluded an outlier of 309 words during the last session. Nine students wrote down more than 50 words during the last session. Among them, one student only wrote 2 words during the first session but 309 words during the last session. These results may indirectly represent that they perceived a positive learning climate while participating in the program.

## Limitations

One limitation of this study was that the proposed program included multiple components. The interpretation of the study results needs to be conducted with caution since it is difficult to differentiate the effect of learning content, course duration, and learning climate or to isolate the contribution of each component. Future studies may consider the separation of cognitive preparation and skill fostering to better identify distinctive attributes and any significant differences among them. Another limitation was the lack of estimating the long-term effect of the intervention. It may be necessary to conduct research that includes longer follow-up periods to track whether the intervention effects among participants were sustained. In addition, there was about 20% attrition in this program. This could be attributed to users of illegal drugs who did not want to reveal themselves. Still, the effect of school climate on the learning environment could vary. Since differences exist in cultural norms and school characteristics, the implementation of the program presented in the present study should take school climate into consideration.

## Conclusion and implications for care

This program provided an alternative approach to prevent the use of illegal drugs among vocational high school students who are experimental drug users. Multiple components were integrated as an early intervention for adolescents’ drug cessation. Although positive climate may be essential for connecting the various components, there are difficulties in quantifying it and conveying it in a structuralized measurement tool. The effectiveness of positive climate may be scientifically verified by comparing three groups: those who receive conventional education, those who receive only the E-course, and those who receive a combination of the E-course and counselor assistance.

To ensure that adolescents who experiment with drugs are treated early, before developing a substance use disorder, school personnel should target them while still at school. Providing one-on-one intervention in schools may not always be feasible or affordable. For reducing the burden of instruction, learning contents could be delivered through an E-course. It is important to encourage school personnel to develop a safe and supportive learning climate and empower them so that they play a vital role in facilitating learning, solving problems, leading discussions, and providing feedback. To extend the connection with students outside the classroom, text messaging using mobile phones is an effective option. Students who were experimental drug users and who have successfully refrained from using drugs can be rewarded for their efforts and accomplishment. Coupon-based incentives, certificates, and awards are strategies that can easily be applied on campus. Since semesters are separated by summer or winter vacation, provisions of booster courses after vacations are necessary for the reinforcement of desired messages and skills.

## Abbreviations

DURSE: Drug Use Resistance Self-Efficacy
LMM: Linear Mixed Model
LST: Life skills Training
PSS: Perceived Stress Scale
