# Correction to: Evaluation of the effects of a designated program on illegal drug cessation among adolescents who experiment with drugs

**DOI:** 10.1186/s13011-019-0231-4

**Published:** 2019-10-30

**Authors:** Chiu-Ching Chang, Jung-Yu Liao, Chiu-Mieh Huang, Hsiao-Pei Hsu, Chih-Che Chen, Jong-Long Guo

**Affiliations:** 1 Department of Health Promotion and Health Education, National Taiwan Normal University, No. 162, Section 1, Heping East Road, 10610 Taipei, Taiwan; 2 Institute of Clinical Nursing, National Yang-Ming University, No. 155, Section 2, Li-Nong Street, 11221 Taipei, Taiwan; 3 Department of Health Promotion and Health Education, College of Education, National Taiwan Normal University, No. 162, Section 1, Heping East Road, 10610 Taipei, Taiwan

**Correction to: Subst Abuse Treat Prev Policy (2018) 13: 2.**

**https://doi.org/10.1186/s13011-017-0139-9**

Following publication of the original article [1], we have been notified that some data in the text should be changed.

Now it reads:

LMMs were used to examine the group differences in patterns of change over time (Table 2). Results of the LMM analysis showed that the intervention group made nonsignificant improvements compared to the comparison group after the main intervention in terms of the scores of stress management (β =2.41, t = 1.81, *p* = 0.073), refusal skills (β =0.61, t = 0.62, *p* = 0.534), pros of drug use (β =0.97, t = 0.38, *p* = 0.703), cons of drug use (β =0.68,t = 0.25, *p* = 0.802) and drug use resistance selfefficacy (β =0.64, t = 0.51, *p* = 0.609). However, after the booster intervention, the participants of the intervention group showed significant improvements compared to their counterparts in the comparison group. There was a significant group × time interaction for the four outcome measures except for cons of drug use (β =3.98, t = 1.45, *p* = 0.150). The intervention group showed an increase in the score of stress management as compared to the comparison group after the booster intervention (β =7.35, t = 5.19, *p* < 0.001). Similar patterns of between-group differences were found in the scores of refusal skills and drug use resistance self-efficacy (β =2.09, t = 2.10, *p* = 0.038; β = 3.47, t = 3.15, *p* = 0.002). The intervention group showed a decrease in the score of pros of drug use as compared to the comparison group after the booster intervention (β = − 7.96, t = − 2.62, *p* = 0.010).

It should read:

LMMs were used to examine the group differences in patterns of change over time (Table 2). Results of the LMM analysis showed that the intervention group made nonsignificant improvements compared to the comparison group after the main intervention in terms of the scores of stress management (β =2.41, t = 1.42, *p* = 0.159), refusal skills (β =0.61, t = 0.56, *p* = 0.577), pros of drug use (β =0.97, t = 0.29, *p* = 0.774), cons of drug use (β =0.68, t = 0.20, *p* = 0.838) and drug use resistance self-efficacy (β =0.64, t = 0.45, *p* = 0.652). However, after the booster intervention, the participants of the intervention group showed significant improvements compared to their counterparts in the comparison group. There was a significant group × time interaction for the four outcome measures except for cons of drug use (β =3.98, t = 1.37, *p* = 0.174). The intervention group showed an increase in the score of stress management as compared to the comparison group after the booster intervention (β =7.35, t = 4.87, *p* < 0.001). Similar patterns of between-group differences were found in the scores of refusal skills and drug use resistance self-efficacy (β =2.09, t = 2.10, *p* = 0.038; β = 3.47, t = 3.09, *p* = 0.003). The intervention group showed a decrease in the score of pros of drug use as compared to the comparison group after the booster intervention (β = − 7.96, t = − 2.41, *p* = 0.017).

## References

[1] Chang, et al. Evaluation of the effects of a designated program on illegal drug cessation among adolescents who experiment with drugs. 2018;13:2. 10.1186/s13011-017-0139-9. 10.1186/s13011-017-0139-9 PMC5771158 29338751

